# Uptake of a fluorescent l-glucose derivative 2-NBDLG into three-dimensionally accumulating insulinoma cells in a phloretin-sensitive manner

**DOI:** 10.1007/s13577-015-0125-3

**Published:** 2015-11-09

**Authors:** Ayako Sasaki, Katsuhiro Nagatomo, Koki Ono, Toshihiro Yamamoto, Yuji Otsuka, Tadashi Teshima, Katsuya Yamada

**Affiliations:** Department of Physiology, Hirosaki University Graduate School of Medicine, 5 Zaifu-cho, Hirosaki, Aomori 036-8562 Japan; Peptide Institute, Inc., Saito Research Center, Ibaraki, Osaka 567-0085 Japan; Graduate School of Science, Osaka University, Toyonaka, Osaka 560-0043 Japan

**Keywords:** l-Glucose, Tumor, Spheroid, Cancer, Biomarker

## Abstract

Of two stereoisomers of glucose, only d- and not l-glucose is abundantly found in nature, being utilized as an essential fuel by most organisms. The uptake of d-glucose into mammalian cells occurs through glucose transporters such as GLUTs, and this process has been effectively monitored by a fluorescent d-glucose derivative 2-[*N*-(7-Nitrobenz-2-oxa-1,3-diazol-4-yl)amino]-2-deoxy-d-glucose (2-NBDG) at the single cell level. However, since fluorescence is an arbitrary measure, we have developed a fluorescent analog of l-glucose 2-[*N*-(7-Nitrobenz-2-oxa-1,3-diazol-4-yl)amino]-2-deoxy-l-glucose (2-NBDLG), as a negative control substrate for more accurately identifying the stereoselectivity of the uptake. Interestingly, a small portion of mouse insulinoma cells MIN6 abundantly took up 2-NBDLG at a late culture stage (≳10 days in vitro, DIV) when multi-cellular spheroids exhibiting heterogeneous nuclei were formed, whereas no such uptake was detected at an early culture stage (≲6 DIV). The 2-NBDLG uptake was persistently observed in the presence of a GLUT inhibitor cytochalasin B. Neither d- nor l-glucose in 50 mM abolished the uptake. No significant inhibition was detected by inactivating sodium/glucose cotransporters (SGLTs) with Na^+^-free condition. To our surprise, the 2-NBDLG uptake was totally inhibited by phloretin, a broad spectrum inhibitor against transporters/channels including GLUTs and aquaporins. From these, a question might be raised if non-GLUT/non-SGLT pathways participate in the 2-NBDLG uptake into spheroid-forming MIN6 insulinoma. It might also be worthwhile investigating whether 2-NBDLG can be used as a functional probe for detecting cancer, since the nuclear heterogeneity is among critical features of malignancy.

**Electronic supplementary material:**

The online version of this article (doi:10.1007/s13577-015-0125-3) contains supplementary material, which is available to authorized users.

## Introduction

Mammalian cells take up d-glucose in a stereoselective manner through plasma membrane transporters, such as GLUTs, whereby only d- and not l-glucose is recognized [1]. We have shown that 2-[*N*-(7-Nitrobenz-2-oxa-1,3-diazol-4-yl)amino]-2-deoxy-d-glucose (2-NBDG) (Online Resource 1a), which was originally synthesized to see the viability of *E. coli* cells [2], is taken up into mammalian cells through GLUTs in a time, concentration and temperature-dependent manner [3, 4]. So far, 2-NBDG has been effectively utilized for monitoring d-glucose uptake in a variety of mammalian cells including pancreatic cells [3, 5], brain cells [6–9], and tumor cells [10–15]. However, since fluorescence is an arbitrary measure, a control substrate has been awaited for more accurately evaluating the GLUT-mediated component [4].

A green fluorescence-emitting 2-[*N*-(7-Nitrobenz-2-oxa-1,3-diazol-4-yl)amino]-2-deoxy-l-glucose (2-NBDLG), the mirror-image isomer of 2-NBDG, was thus developed as the control substrate (Online Resource 1b) [16, 17]. For evaluating an occurrence of non-specific uptake such as due to a loss of membrane integrity, we found it strongly helpful to use 2-NBDLG simultaneously with a membrane-impermeable l-glucose derivative 2-TRLG, which bears a large red fluorophore Texas red (Online Resource 1c) [17].

To explore the stereoselective uptake of mammalian cells with such fluorescent tracers, we have used mouse insulin-secreting clonal (MIN6) cells [18]. Surprisingly, when MIN6 cells cultured over 10 days in vitro (DIV) were examined, the fluorescence of cells increased significantly not only by application of 2-NBDG, but also by 2-NBDLG. In the present study, we characterize unique features of the 2-NBDLG uptake into the insulinoma cells.

## Methods

### Confocal microscopic study

#### Culture

MIN6 cells were grown, according to the original protocol in Dulbecco’s modified Eagle’s medium (DMEM) (D5648, Sigma-Aldrich) [18]. Only cells in earlier passages (from 5 up to 10 times) were used in the present study except for Online Resource 2 [19]. On the day of culture, poly-l-lysine hydrobromide (PLL) (P6282, final concentration 1/500, Sigma)-coated, small glass coverslips (No. 0, Matsunami) were placed on 35 mm non-coated dish (Iwaki). Cells for the confocal measurement were seeded at a density of 1000 cells per cover slip. Culture medium was half exchanged every 3 days.

#### Measurement

The tracer application and image acquisition were conducted by modifying the method reported previously [4, 17]. In brief, a temperature-controlled custom made chamber was placed on a motor-driven xyz stage of a laser confocal microscope (TCS-SP5, Leica). 2-NBDG/2-NBDLG and 2-TRLG were excited by a single 488 nm laser source and the fluorescence was detected in 500–580 nm and 580–740 nm wavelength range, respectively, with a dichroic mirror at 500 nm (RSP 500). 4′,6-diamidino-2-phenylindole (DAPI) was applied for nuclear staining in live-cell condition at 37 °C. An objective lens HCX PL APO 40x/1.25-0.75 OIL was used except for Online Resource 2 and 7, for which HC PL APO 20x/0.70 IMM was used.

### Fluorescence microplate reader experiments

#### Culture

MIN6 cells were seeded at a density of 6000 cells/well on 96-well clear-bottom plate (µClear-plate #655090, Greiner Bio-One). Wells in columns 3 and 5 (rows from B to F, total 12 wells) were used for culture and no cell was seeded in the top (A) and the bottom (H) rows in these columns. Typically, 10 μl of cell suspension was plated at 6 × 10^5^ cells/ml on the center of each well, and left for 20 min in CO_2_ incubator at 37 °C. 200 μl of DMEM was then added to each well. Cells incubated for 10-14 DIV were used for measurement.

#### Measurement

For the measurement of the tracer uptake, a fluorescence microplate reader was used with its operation software (FlexStation and SoftMax Pro, Molecular Devices). The fluorescence was measured three times from the bottom of the plate and was averaged. Excitation, emission, and cutoff wavelength were 470, 540, and 495 nm, respectively.

Just before measurements, culture medium was removed from each well leaving 50 µl. Cells were then washed five times with 200 µl of standard Krebs–Ringer Buffer Solution (KRB) (in mM; 129 NaCl, 4.8 KCl, 1.2 KH_2_PO_4_, 1.2 MgSO_4_, 1.0 CaCl_2_, 10 HEPES, 5.0 NaHCO_3_, 5.6 d-glucose, 0.1 carbenoxolone, pH 7.30–7.35) at room temperature (26 ± 1 °C). After the fifth wash, KRB was added to adjust the height of solution to that of blank wells in column 4, in which 200 µl of KRB without containing tracers was added. Nine regions of interest (ROIs, 1.5 mm in diameter, each contained typically ~5000 cells or more when measured at 12 DIV) were preset in single wells of 96-well plate. By visualizing cells before and after the experiment with a flatbed scanner (GT-X820, Seiko Epson), ROIs, in which cells were unevenly seeded or lost during washout, were excluded from the analysis.

Autofluorescence was measured for individual ROIs. According to a precisely timed protocol, 50 μl of 2-NBDG- or 2-NBDLG-containing KRB solution (400 μM) was then added into 8 wells from A to H in column 3, in which 50 μl of KRB was pre-loaded, using a calibrated 8-channel pipette (final concentration, 200 μM). 30 s later, fluorescent tracers were similarly added to wells in column 5. The top (A) and bottom (H) wells in columns 3 and 5 were used as control to check that the tracer was successfully washed out from the solution. After adding the tracer solution, the plate was quickly placed on the tray in FlexStation at 37 °C. Five minutes later, 50 μl of the tracer solution was removed from wells in column 3, and 300 μl of KRB solution was added at room temperature. Thirty seconds later, similar washout process was done in column 5. After repeating this process 7 times, 300 µl of KRB was finally added and 150 µl of KRB was removed; the fluorescence was then measured and compared among ROIs. Transient increase in fluorescence due to a loss of membrane integrity decreased to a large extent during this washout procedure.

For Na^+^-free experiments, NaCl in KRB was replaced by choline chloride. For glucose competition assay, 50 mM of d- or l-glucose solution was prepared by reducing NaCl so that the osmolality of the solution was identical to that of control.

### Reagents

2-NBDLG (23003-v, Peptide Institute), 2-NBDG (23002-v, Peptide Institute), and 2-TRLG were provided by Peptide Institute Inc. and used as described previously [17]. Carbenoxolone (C4790, Sigma) was used to block gap junction/hemi-channel. Phloretin (P7912, Sigma), cytochalasin B (C6762, Sigma), and 4,6-*O*-ethylidene-d-glucose (E0402, Tokyo Chemical Industry) were applied prior to the tracer application. DAPI (D523, Wako) was added in some experiments for nuclear staining. pSIVA-IANBD (IMGENEX) and propidium iodide (IMGENEX) were used as a polarity-sensitive, real-time marker for apoptotic cells and an indicator for necrotic cells, respectively, according to the manufacturer’s instruction. NBD-NH_2_ was synthesized by the reaction of NBD-F and ammonia. NBD-OH was obtained as a by-product on the synthesis of 2-NBDLG. Both NBD-NH_2_ and NBD-OH were used at a final concentration of 200 µM.

### Statistics

ANOVA and Bonferroni–Dunn test were used. Error bars represent SD.

## Results

### Imaging of 2-NBDG and 2-NBDLG uptake with confocal microscopy

**Fig. 1:**
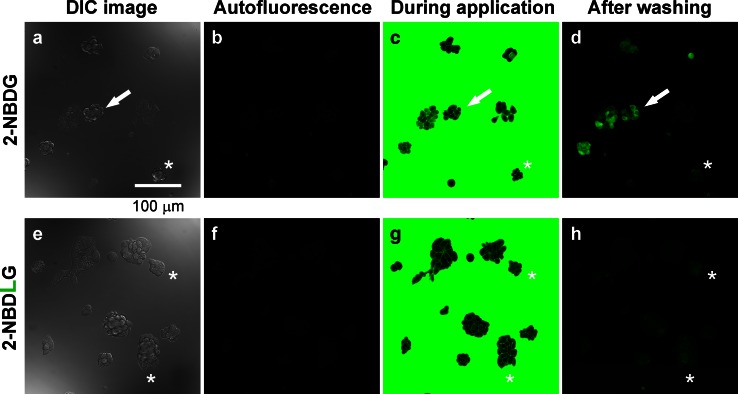
Representative images of MIN6 cell spheroids subjected to a brief application of solution containing either 2-NBDG (**a**–**d**) or 2-NBDLG (**e**–**h**) at 6 days in vitro (DIV). **a** Differential interference contrast (DIC) image. **b** Autofluorescence before application of the fluorescent tracer. **c** During application of 100 μM of 2-NBDG-containing solution for 3 min. **d** Fluorescence image taken at 4 min after starting washout of the tracer. 2-NBDG uptake varied considerably from prominent (*arrow*) to minimal (*asterisk*) among cells. **e–h** Similar to **a**–**d** but for application of 100 μM of 2-NBDLG to the same series of culture examined on the same day. The uptake of 2-NBDLG into MIN6 cells at this stage was very weak, although some cells showed faint fluorescence (*asterisks*). *Scale bar* is common to all images

MIN6 cells, when seeded at a very low density (1000 cells per coverslip), formed small three-dimensional spheroid after several days (Fig. 1a, e). At 6 DIV, a brief superfusion with 100 μM of 2-NBDG solution for 3 min followed by washout increased the cellular fluorescence in varying intensity among cells (Fig. 1b–d). Such unique heterogeneity in the 2-NBDG uptake is very different from relatively homogeneous uptake of the tracer in two-dimensionally spreading MIN6 cells (Online Resource 2). By contrast, no such increase in the fluorescence was detected by similarly applying 100 μM of 2-NBDLG (l-form isomer) solution (Fig. 1e–h), although one may notice cells showing faint fluorescence if examined closely (asterisks in Fig. 1e–h).

**Fig. 2:**
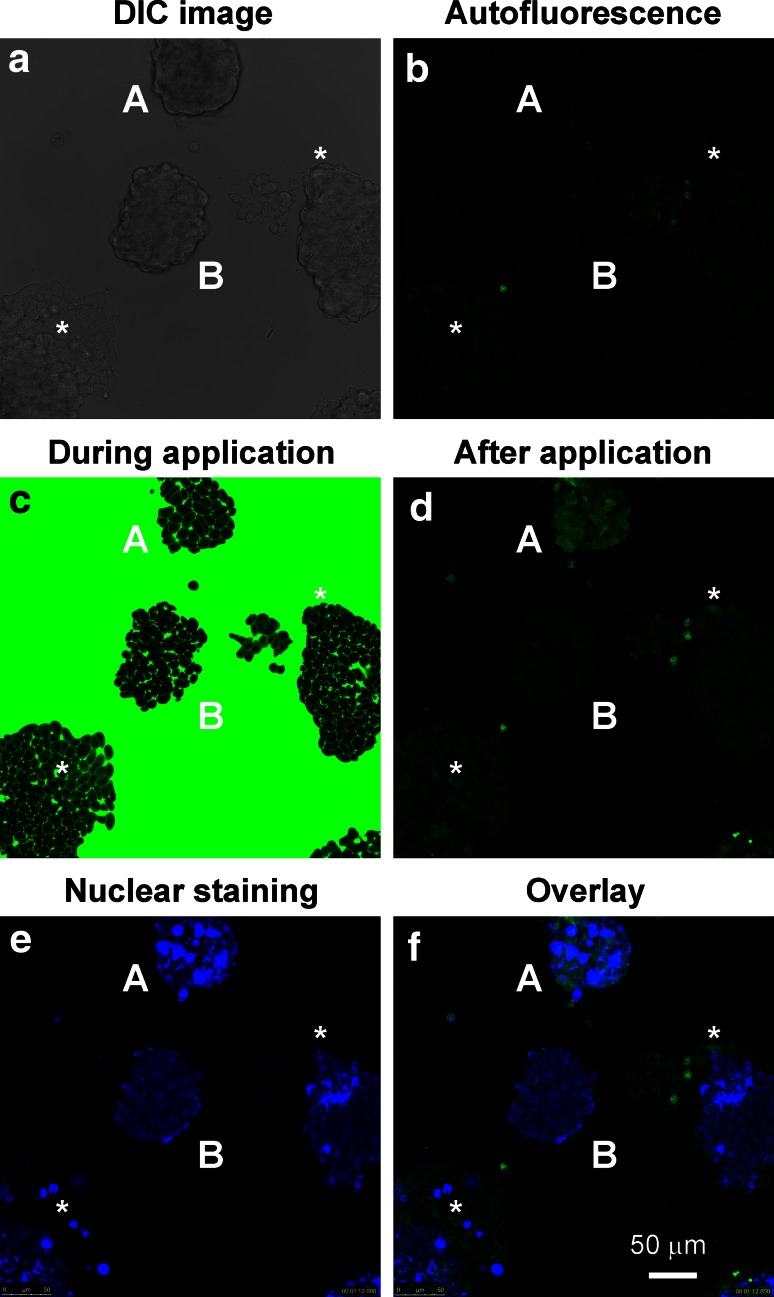
Uptake of 2-NBDLG into MIN6 cells forming spheroids. **a** DIC image of MIN6 cells at 11 DIV. **b** Autofluorescence measured before application of 100 μM of 2-NBDLG for 3 min. **c** During application of 2-NBDLG. Note that both spheroids (*A* and *B*) were evenly superfused by 2-NBDLG solution. **d** Two minutes after starting washout of the 2-NBDLG solution. Cells in the *upper spheroid* (*A*) exhibited a strong 2-NBDLG fluorescence in the cytosol, whereas only a minimum increase in the fluorescence was seen in cells in the *lower spheroid* (*B*). **e** Live-cell nuclear staining by DAPI, conducted after finishing 2-NBDLG application. *Upper spheroid* contained cells having extremely DAPI-positive large nucleus (*A*), whereas lower one showed nuclei of normal size. **f** Merged image of **d** and **e**. Multi-stack z-sections of DAPI staining are available in Online Resource 4 in detail. Asterisks indicate cells moderately positive for 2-NBDLG in other spheroids. *Scale bar* is common to all *panels*

Interestingly, more drastic 2-NBDLG uptake was detected at 10–15 DIV, when much thicker (>50 μm-thick) spheroids were grown (A in Fig. 2). Of two similarly shaped MIN6 spheroids (A and B in Fig. 2a), only upper one showed remarkable 2-NBDLG uptake (Fig. 2b, d), whereas lower one increased fluorescence only slightly.

Nuclear staining with DAPI in live-cell condition further revealed that the upper spheroid consisted of heterogeneous cells, including large cells which bear unusually large and strongly DAPI-positive nucleus as well as small cells having nucleus of ordinary size (Fig. 2e, f; see also Online Resource 4). By contrast, the lower spheroid consisted of cells bearing evenly sized small nuclei that were only weakly positive for DAPI (B in Fig. 2e, f).

It is noteworthy that 2-NBDLG was taken up not only into such large presumably multinucleated cells but also into small cells having nucleus of ordinary size (Fig. 2d, f). DAPI-positive, fibrous processes were seen in the upper spheroid, in addition (Online Resource 4) [20]. Similar fibrous processes appeared in other clusters that occasionally took up 2-NBDLG (asterisks in Fig. 2e and Online Resource 4).

### Quantitative evaluation of the 2-NBDG and 2-NBDLG uptake by a fluorescent microplate reader

Thick and large spheroids easily collapsed sometimes in hours. Moreover, whether or not spheroids of interest would take up abundant 2-NBDLG was difficult to be expected prior to imaging. Thus, we evaluated the average fluorescence intensity of a large number of MIN6 cells subjected to 2-NBDLG compared with those subjected to 2-NBDG at 10–14 DIV using a fluorescent microplate reader.

**Fig. 3:**
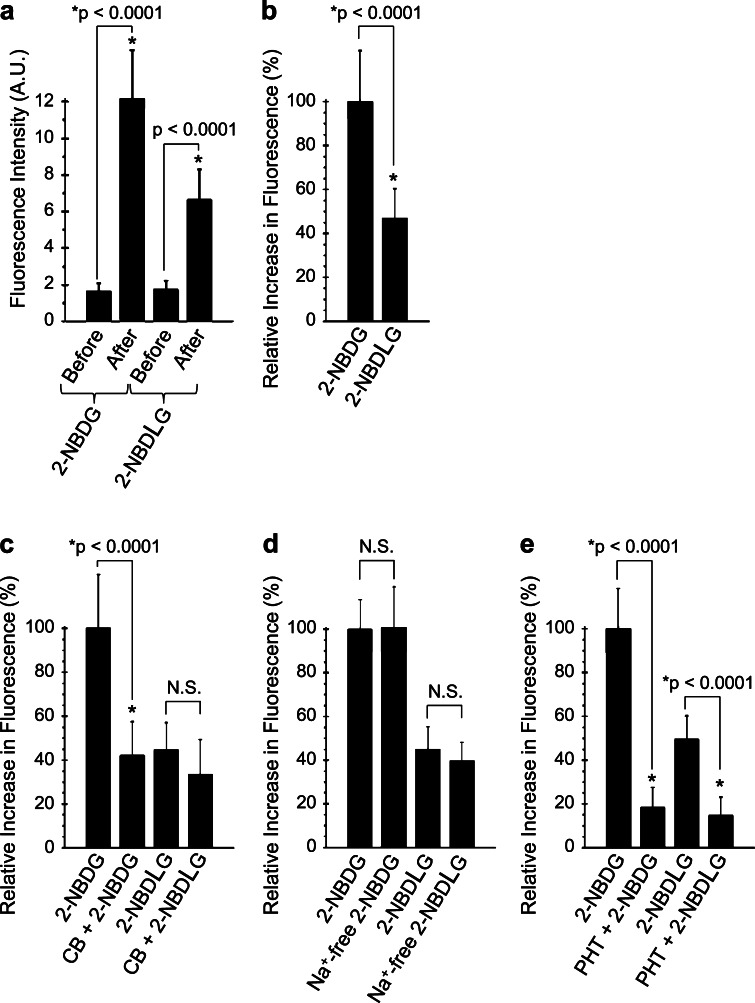
Quantitative evaluation of the 2-NBDG and 2-NBDLG uptake into MIN6 cells examined at 10-13 DIV. **a** Changes in the fluorescence of MIN6 cells subjected to 2-NBDG or 2-NBDLG solution. **b** The net increase in the fluorescence in (**a**). **c** Increase in the fluorescence in the absence or presence of a GLUT inhibitor cytochalasin B (10 μM, CB). **d** Effect of Na^+^-free condition on the 2-NBDG and 2-NBDLG uptake. **e** Effect of phloretin (150 μM, PHT) on the 2-NBDG and 2-NBDLG uptake. Values are expressed as mean percent increase in the fluorescence relative to the fluorescence increase for 2-NBDG application on the same 96-well plate (**b**–**e**). Values in individual columns represent mean fluorescence of more than 12 ROIs (more than 5000 cells are included in each ROI) and expressed as mean ± SD

When 200 μM of 2-NBDG was applied for 5 min, the average fluorescence intensity of ROIs increased from 1.6 ± 0.4 arbitrary unit (AU, autofluorescence) to 12.2 ± 2.6 AU (*n* = 48, *p* < 0.0001, Fig. 3a). 2-NBDLG, applied simultaneously but to other wells, also noticeably increased the fluorescence from 1.7 ± 0.5 to 6.7 ± 1.6 AU (*n* = 51, *p* < 0.0001, Fig. 3a). Measurements were performed in quadruplicate and the ratio of the net increase in the fluorescence for 2-NBDLG to that for 2-NBDG was 44.9 ± 1.7 % in average (Fig. 3b).

Cytochalasin B, which acts as a GLUT inhibitor when used at a low dose (10 μM), significantly decreased the 2-NBDG uptake into MIN6 cells (*p* < 0.0001, Fig. 3c). 2-NBDG uptake was decreased by 61.9 ± 3.9 % on average in the presence of cytochalasin B in experiments performed in triplicate. On the other hand, 2-NBDLG uptake on the same plates was attenuated only slightly by 20.5 ± 9.6 % in the presence of cytochalasin B (Fig. 3c). Interpreted another way, in the presence of cytochalasin B, the substantial component remained in the uptake of 2-NBDLG, and that of 2-NBDG as well, implicating an involvement of non-GLUT mechanisms for both uptake of the l- as well as d-derivatives.

Consistently, a large amount of d-glucose (50 mM) reduced the 2-NBDG uptake only moderately by 28.9 ± 12.4 % (*p* < 0.0001, Online Resource 3a), and no inhibition was detected by the same amount of l-glucose. Similar to Fig. 3c, 2-NBDLG uptake was attenuated only slightly by 50 mM d-glucose (Online Resource 3b). The same amount of l-glucose showed no effect on the uptake. An involvement of SGLTs, energy-demanding Na^+^-coupled glucose transporters, is unlikely, since both 2-NBDLG and 2-NBDG uptake persisted in the absence of Na^+^ ion in the extracellular solution (Fig. 3d).

To our surprise, 150 μM of phloretin, a broad spectrum inhibitor against membrane transport including GLUTs/water channels [1, 21], virtually abolished the increase in the fluorescence for 2-NBDLG as well as for 2-NBDG application, leaving only minimally detectable fluorescence (*p* < 0.0001, Fig. 3e). Experiments were performed in quadruplicate and similar results were obtained. It is unlikely that the 2-NBDLG fluorescence was produced by fluorophore NBD, since the fluorescence of MIN6 cells was barely detectable when subjected to KRB containing either NBD-NH_2_ or NBD-OH (data not shown).

### Cellular heterogeneity in spheroids revealed by a combined use of 2-NBDLG and 2-TRLG

**Fig. 4:**
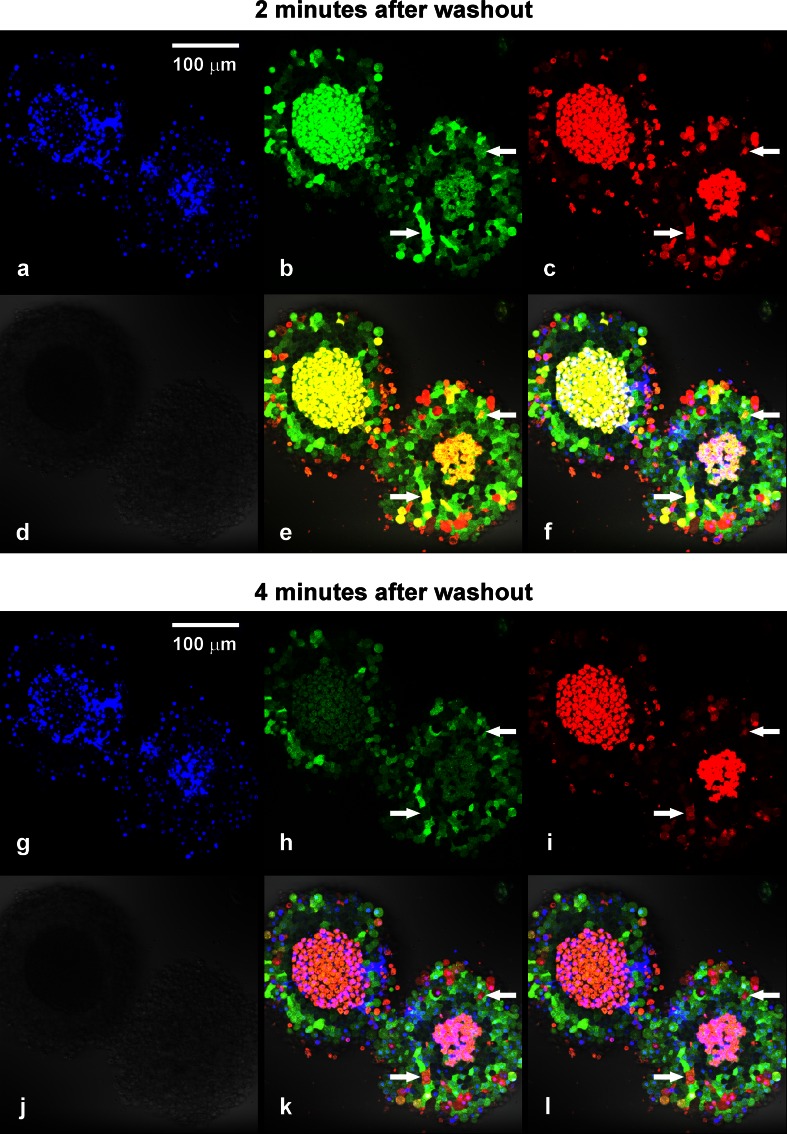
Confocal microscopic images of 12 DIV MIN6 spheroids subjected to 100 μM of 2-NBDLG (*green*) and 20 μM of 2-TRLG (*red*) mixture for 3 min followed by washout. **a** Nuclear staining with DAPI in live cell condition. The central core region of spheroids appears to be necrotic (see also **d**). **b** and **c** Fluorescence images taken at 2 min after starting washout of the tracers in the *green* (**b**, 500–580 nm) and the *red* (**c**, 580–740 nm) channel, reflecting entrance of 2-NBDLG and 2-TRLG, respectively. **d** Differential interference contrast (DIC) image. **e** Overlay of the *green*, *red*, and DIC images. **f** Overlay of (**a**) and (**e**). Cellular heterogeneity is clearly seen by a combination of the two fluorescent colors. Cells indicated by *arrows* exhibited *yellow color* at 2 min (**e**), turned *red* at 4 min (**k**). This is because *green* 2-NBDLG was lost (**b**, **h**), while *red* 2-TRLG remained (**c**, **i**). If one saw a single 2-NBDLG image (**b**), cells indicated by *arrows* would have been misinterpreted to be similar to cells nearby. **g–l** Similar to **a**–**f** but images taken at 4 min after starting washout. Numbers of *green* cells with no *red* fluorescence, seen in the area surrounding the central core, preserved their color for at least up to 30 min (**h**, **i**, **k**). Also noted are *dark cells* in the area just surrounding the central core (**b**, **e**, **h**, **k**). *Bars* are common to all *panels*

Tumor cells might internalize a wide variety of compounds if exposed for many minutes [22, 23]. To evaluate an occurrence of non-specific uptake, the second l-glucose derivative 2-TRLG [17] was applied simultaneously with 2-NBDLG (Fig. 4; Online Resource 1). 2-TRLG is a unique “membrane-impermeable” l-glucose derivative bearing Texas Red emitting red fluorescence [17]. As illustrated, combined administration of 2-NBDLG (100 μM) and 2-TRLG (20 μM) for 3 min to well-developed MIN6 spheroids (12 DIV) revealed spatially and temporally heterogeneous uptake among cells (Fig. 4). At 2 min after starting washout of the tracers, cells located in the central core of the spheroids turned yellow in merged image (Fig. 4e), indicating massive entrance of 2-NBDLG and 2-TRLG (Fig. 4b, c). However, most of these cells had lost the yellow color by 4 min after washout (Fig. 4k) due to a rapid exit of 2-NBDLG (Fig. 4h), while maintaining 2-TRLG intracellularly (Fig. 4i).

On the other hand, a considerable number of cells in the area surrounding the central core showed varied strength of green fluorescence, which well persisted at 4 min after washout (Fig. 4b, h, see Online Resource 5). The spatial distribution of such green cells largely overlapped with the area containing cells bearing only weakly DAPI-positive nuclei (Fig. 4a, g, f and l), and with non-apoptotic/non-necrotic region (Online Resource 7), suggesting that such 2-NBDLG-positive/2-TRLG-negative cells represent viable cells.

Cells showing pale red, deep red, and yellow color were distinguished in the surrounding area at 2 min (Fig. 4e). These fluorescent colors can be used as a measure reflecting loss of membrane integrity from severe to less severe in this order and, indeed, some deep red cells lost their color at 4 min. Dark quiescent cells were found in peri-central region, which took up little 2-NBDLG as if they were normal cells (Fig. 4e, k). Similar cells could be found as well when d-glucose derivative 2-NBDG was applied similarly with 2-TRLG, appeared less prominent though (Online Resource 6). Further quantification of such dark cells was not conducted in the present study, because only a small portion of tight and thick MIN6 spheroids demonstrated such typical profile of uptake.

## Discussion

In the present study, we have shown that a small portion of insulin-secreting clonal cells (MIN6) took up abundant 2-NBDLG when they formed three-dimensional, multi-cellular spheroids exhibiting heterogeneous nuclei at a late culture stage. The 2-NBDLG uptake occurred specifically in a phloretin-inhibitable manner. A combined use of 2-NBDLG with a membrane-impermeable, red fluorescence-emitting l-glucose derivative 2-TRLG provides a unique method for visualizing heterogeneity of tumor cells in multiple colors.

### Characterization of tumor cells by fluorescent l-glucose derivatives

Noticeable uptake of 2-NBDLG proceeded along with formation of three-dimensional spheroids, consisting of cells bearing nuclei of heterogeneous size accompanied by DAPI-positive fibrous processes in some cases. Such features are among major cytological criteria in clinical diagnosis for tumor cells suspected of high grade of malignancy.

Multi-cellular spheroids are thought to emulate the cellular heterogeneity in tumor typically found in the body cavity fluid and in solid tumors [24, 25]. In such three-dimensionally grown tumor, there are cells in intermediate states between perfectly healthy and totally collapsed, due to insufficient oxygen/fuel supply and metabolite clearance, pharmacological treatment, and inflammatory response. The existence of such intermediate cells makes it often difficult to characterize tumor cells using functional probes such as 2-NBDG/2-NBDLG.

2-TRLG can be used as a sensitive measure for the membrane state. Once 2-TRLG entered into the cytosol of such intermediate cells, it remained intracellularly for tens of minutes, whereas it disappeared soon when permeated into totally collapsed cells (Fig. 4). Since a more hydrophilic analog of 2-TRLG failed to show such characteristics (data not shown), charged lipophilic property of 2-TRLG might well be related to the retention. Such unique feature of 2-TRLG is useful for recognizing partially damaged cells during live imaging, contrasted to a commercially available dead cell marker such as propidium iodide, which irreversibly binds to the nucleus once entering into cells (Online Resource 7).

Interestingly, there were cells sustaining 2-NBDLG fluorescence for over 30 min as well as dark and quiescent cells in the area surrounding the necrotic central core of spheroids. Questions are posed what the difference between 2-NBDLG-positive and negative cells is, and whether 2-NBDLG is metabolized after entering into tumor cells.

### Mechanistic consideration underlying 2-NBDLG uptake into tumor cells

From the fact that neither cytochalasin B nor a large amount of d-glucose could totally abolish the uptake of 2-NBDLG and of 2-NBDG, it may be speculated that MIN6 insulinoma expresses phloretin-sensitive, but non-GLUT, non-stereoselective pathways when forming multi-cellular spheroids. Glucose entry into cells might well occur not only through saturable (carrier-mediated) processes requiring glucose binding to the postulated recognition site of the transporter like GLUTs [26, 27] but also through non-saturable ones. Indeed, use of 2-NBDG has suggested in some plant cells that a mercury-sensitive, water-channel-like mechanisms operate concomitantly with saturable processes [28]. 2-NBDLG uptake into MIN6 cells was virtually abolished by phloretin, which is the aglycone of phlorizin, a phytoalexin produced by such as apple and cherry trees. Phloretin, being used as a GLUT inhibitor for many years, is also known as a potent inhibitor of aquaporins [21]. Thus, it is of interest to examine if such channel-like processes participate in the glucose transport in insulinoma cells [21, 29].

A large amount of 4,6-*O*-ethylidene-d-glucose has been used by some investigators as a competitive inhibitor against GLUT-mediated 2-NBDG transport, postulating that it selectively interacts with the outward-facing sugar binding site of a carrier [9]. However, 4,6-*O*-ethylidene-d-glucose might not fully reflect the stereoselective property of d-glucose, since its mirror-image isomer also significantly inhibited 2-NBDG uptake into MIN6 cells (data not shown).

### Clinical significance of the uptake of fluorescent l-glucose derivatives

Evidence has been accumulated which shows that 2-NBDG is taken up into variety of tumor cell lines, such as derived from melanoma, breast, liver, colorectal cancers, and glioma [10, 12, 22, 27]. 2-NBDG has also been used as a contrast agent for imaging biopsy specimen obtained from oral, breast, and Barrett’s esophagus cancer patients [11, 13, 14]. However, critical issues to be solved when using d-glucose derivatives in tumor imaging may include how to reduce the uptake into normal tissues containing fat and muscle, and how to discriminate the uptake into tumor cells from that due to inflammatory and/or non-cancerous anomalies [12, 14]. Difficulty in applying them for diabetic patients may also be pointed out. The least interaction with GLUTs, if any, of l-glucose derivatives like 2-NBDLG would make undesirable binding to non-tumor cells, or toxic effect in other words, minimum compared to that expected when using 2-NBDG.

One of the key therapeutic strategies against rapidly growing tumor would be deprivation of energy source and nutrients [30]. However, tumor cells that survive in low-nutrient environment might well develop regulatory mechanisms enabling utilization of unusual carbohydrate as a carbon source as reported in some lower organisms [31, 32]. Heterogeneous nuclei and tubulin-like fibrous structure are among important features of malignant tumor spheroids [33]. Illuminating the breakdown in the stereoselectivity of tumor cells by their heterogeneous acceptance of unnatural sugar/sugar analogs might serve a new indication for cancer diagnosis eventually at the single cell level and help determining therapies against cancer. Further investigation is required to clarify the transport mechanism, intracellular fate, as well as limitation of 2-NBDLG in tumor imaging.

## Electronic supplementary material

Supplementary material 1 (PDF 1371 kb)
