# Supplementary material for: Uptake of a fluorescent l-glucose derivative 2-NBDLG into three-dimensionally accumulating insulinoma cells in a phloretin-sensitive manner
Source: Hum Cell. 2015 Nov 9;29:37–45. doi: 10.1007/s13577-015-0125-3 (PMC4705143; doi:10.1007/s13577-015-0125-3)
Supplement: Supplementary file 1 — Supplementary material 1 (PDF 1371 kb) [file 13577_2015_125_MOESM1_ESM.pdf]

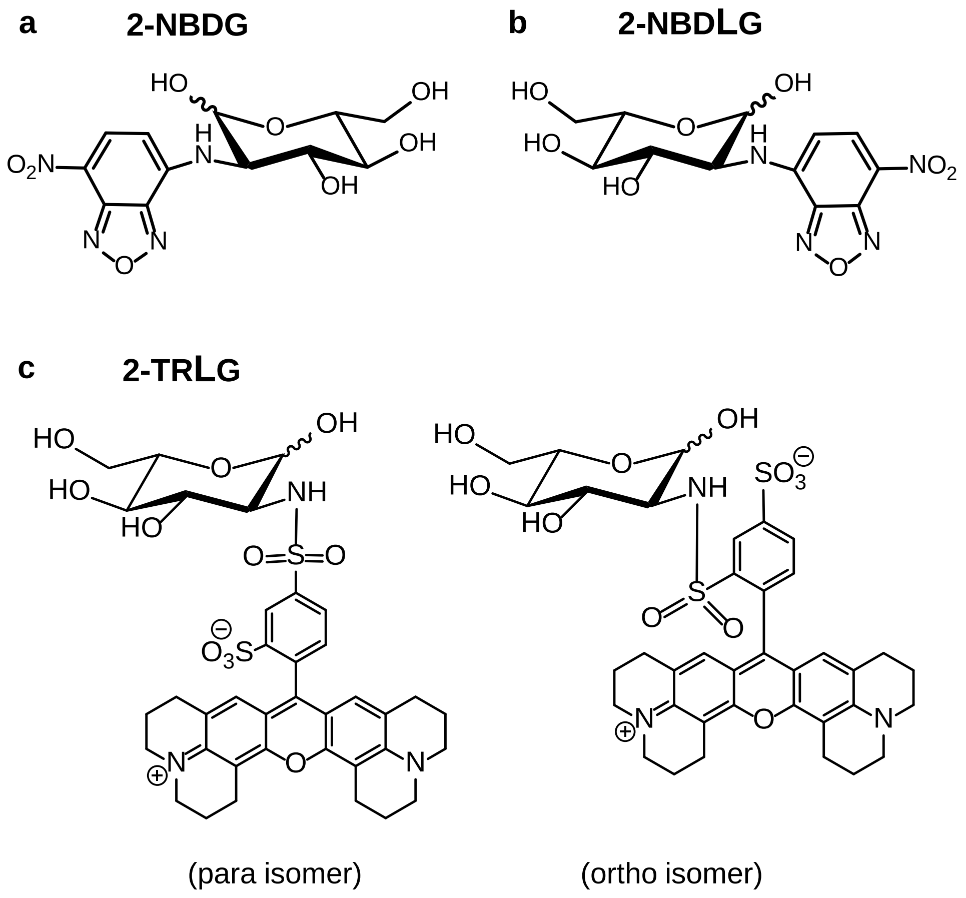

Online Resource 1. Chemical structures of 2-NBDG (a), 2-NBDLG (b), and 2-TRLG (c), respectively.

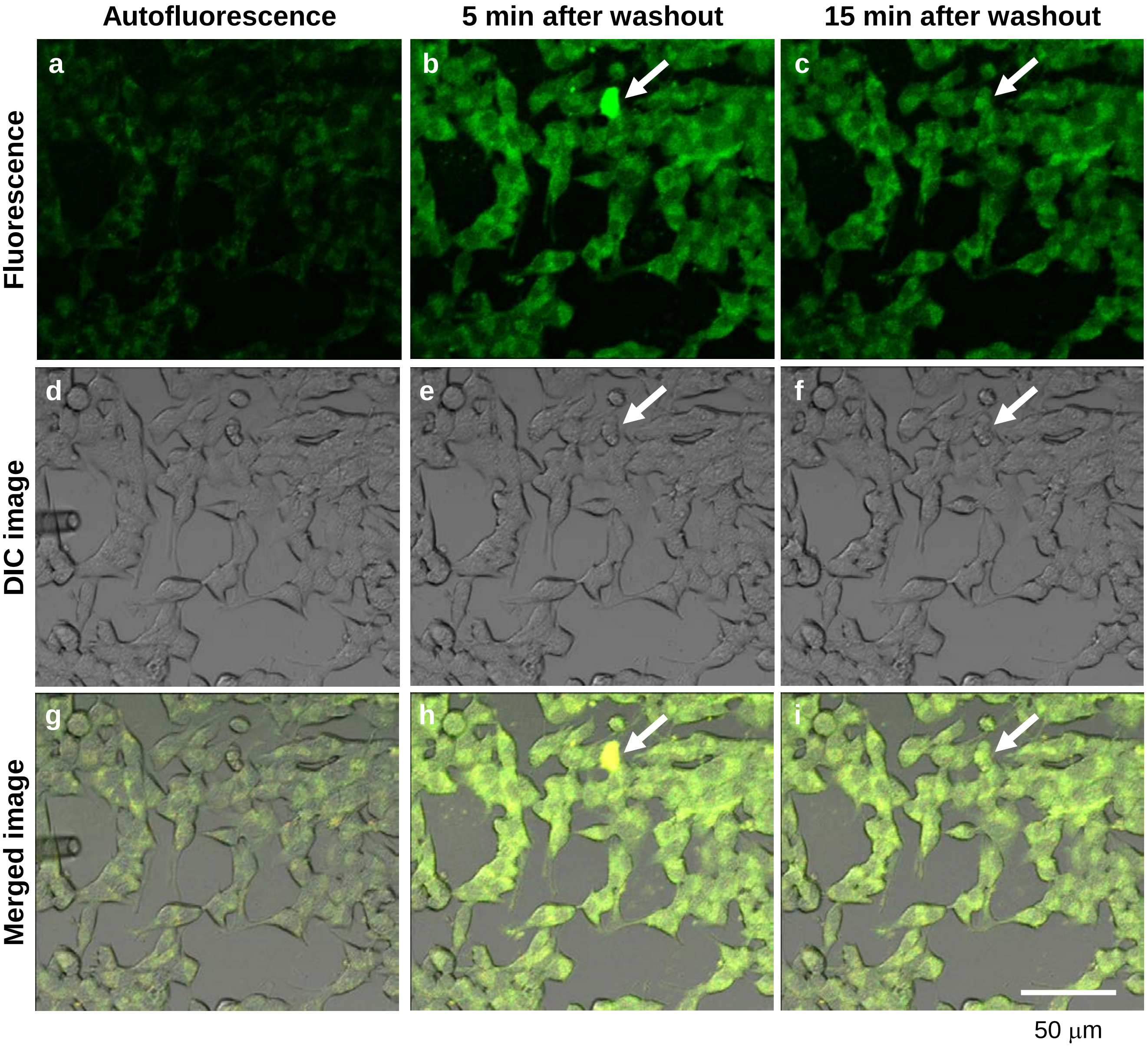

Online Resource 2. Typical uptake of 2-NBDG into two-dimensionally spreading MIN6 cells (passages, possibly over 40 times). Representative fluorescence images (a-c), differential interference contrast (DIC) images (d-f), and merged images (g-i) are shown. **a**, Autofluorescence before application of 200 μM of 2-NBDG for 1 minute. **b** and **c**, Fluorescence images taken at 5 minutes and 15 minutes after starting washout of 2-NBDG solution, respectively. Note that most cells exhibit homogeneous fluorescence except for a cell indicated by an arrow, which showed a strong fluorescence at 5 minutes, and lost the fluorescence intensity at 15 minutes, possibly by leaking out of 2-NBDG due to a loss of membrane integrity. A glass pipette seen in the left was used to apply the fluorescent tracer in this experiment.

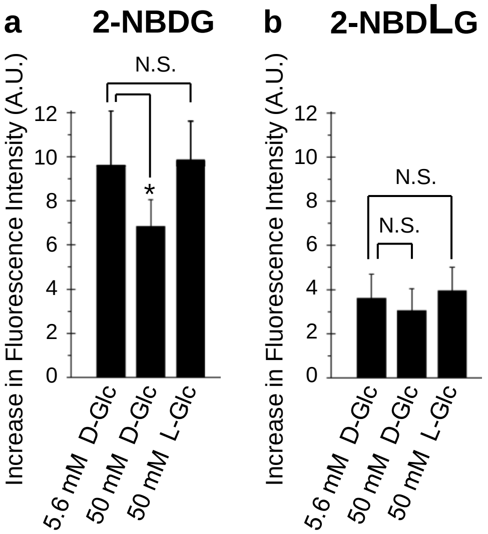

Online Resource 3. Effect of a large amount of D- or L-glucose on the uptake of 2-NBDG (a) and 2-NBDLG (b) into MIN6 cells examined at 10-12 DIV. **a**, 2-NBDG uptake expressed as the increase in fluorescence intensity was reduced only moderately by 50 mM of D-glucose ($p < 0.0001$), and no reduction was detected by the same amount of L-glucose. Experiments were performed in triplicate, and the mean increase in the fluorescence for 2-NBDG application in the presence of either 50 mM of D- or L-glucose to that in control solution containing 5.6 mM of D-glucose were 70.9 ± 0.2 % or 104.2 ± 2.3 %, respectively. **b**, Similar to (a), but for 2-NBDLG application. The uptake of 2-NBDLG was attenuated only slightly by 50 mM of D-glucose, while majority of the fluorescence remained (81.5 ± 7.2 % in average) in experiments performed in septuplicate. No significant change in 2-NBDLG uptake on the same plates was detected by 50 mM of L-glucose (102.1 ± 3.6 %). Values represent mean fluorescence of more than 33 ROIs, and are expressed as mean ± S.D.

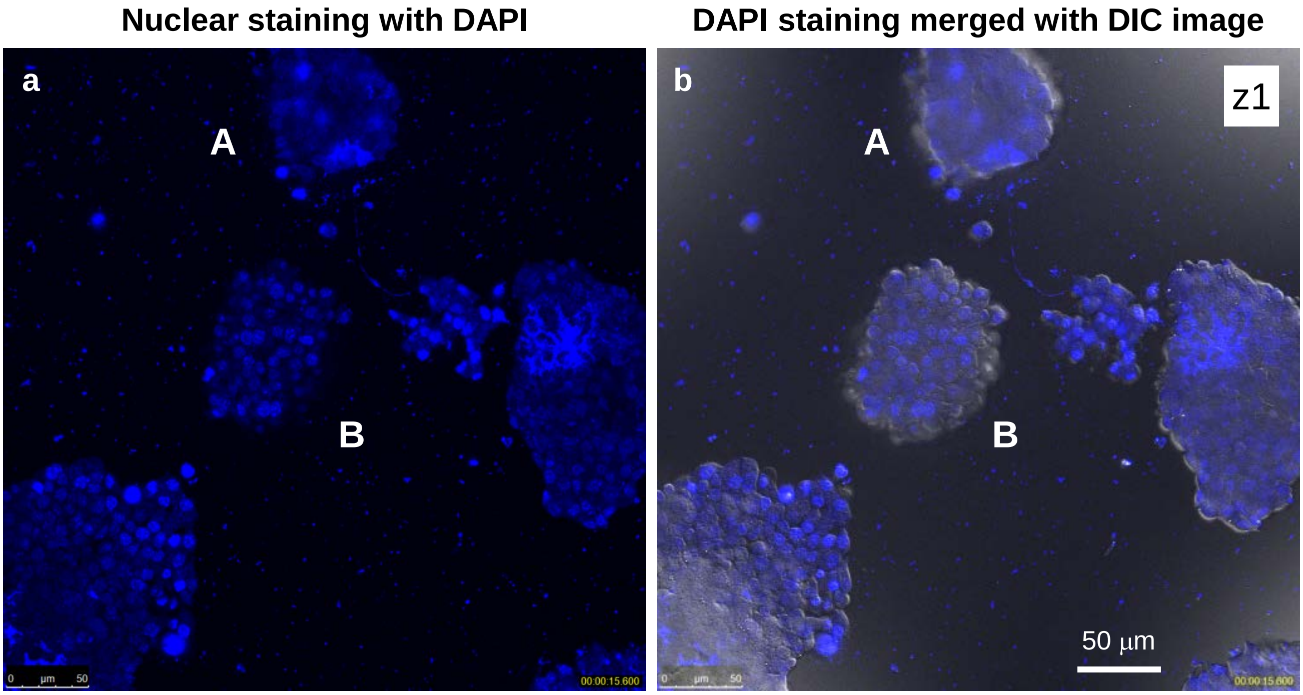

Online Resource 4-1. Serial DAPI and DIC images (z-stack) for Figure 2. The number in the upper-right corner indicates z position, which was numbered consecutively from the cover slip surface every 6 microns. **a**, DAPI staining. **b**, Overlay of DAPI and DIC images. Application of DAPI was done for living cells at 37ºC for an hour (see text). Spheroid A is characterized by strongly DAPI-positive large nuclei (see z2-z5), whereas spheroid B consisted of cells having minimally stained nuclei of ordinary size (see z3-z6) except for necrotic cells (z1 and z2). Scale bar is common to all panels.

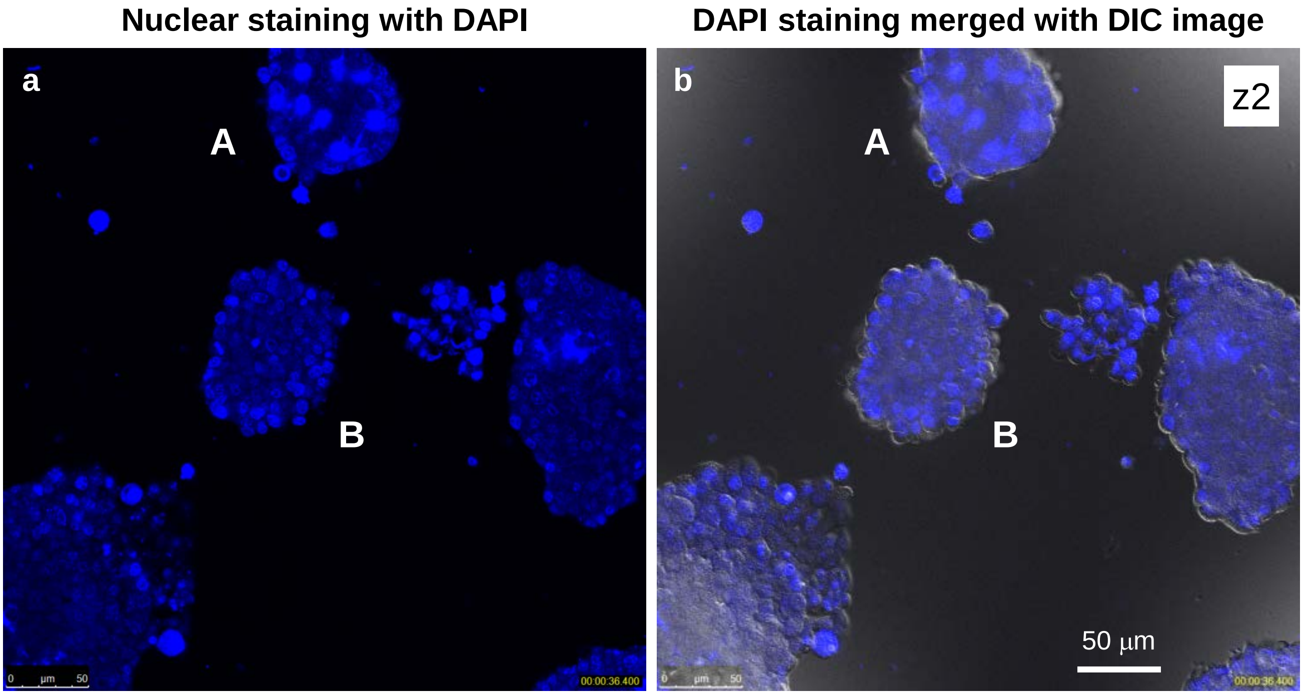

Online Resource 4-2. Similar to Online Resource 4-1, but image taken at 6 microns above.

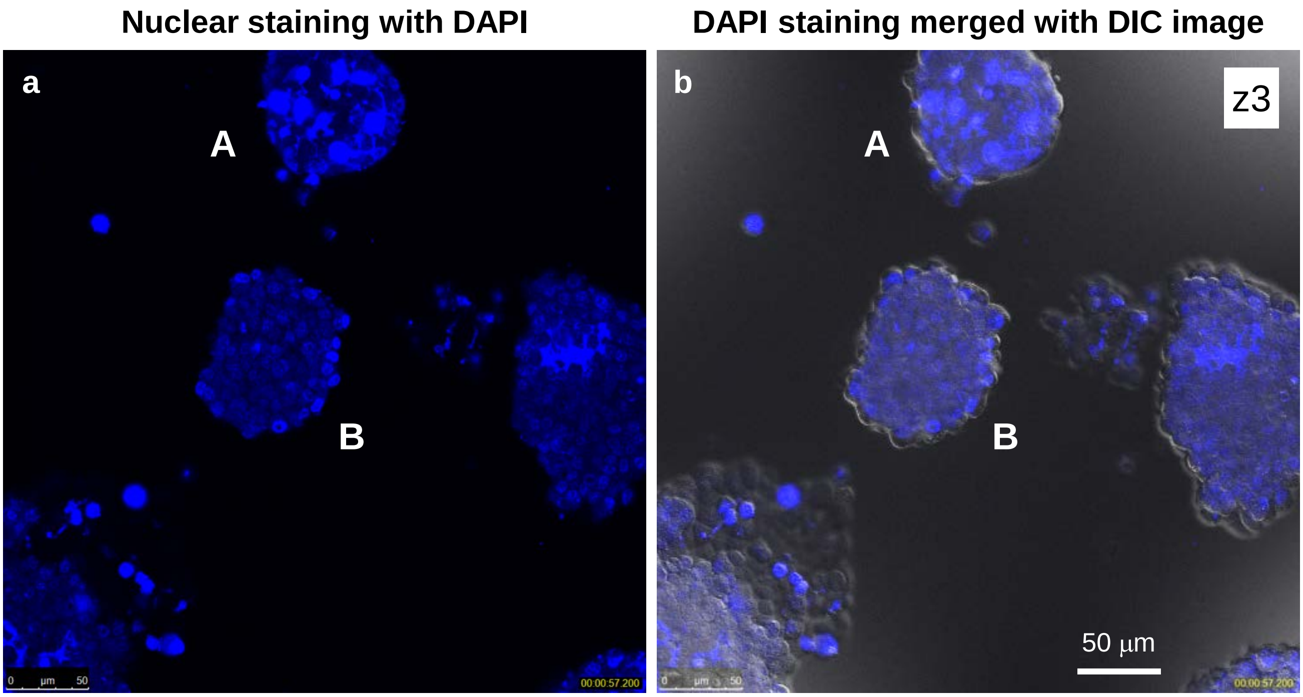

Online Resource 4-3. Similar to Online Resource 4-1, but image taken at 12 microns above.

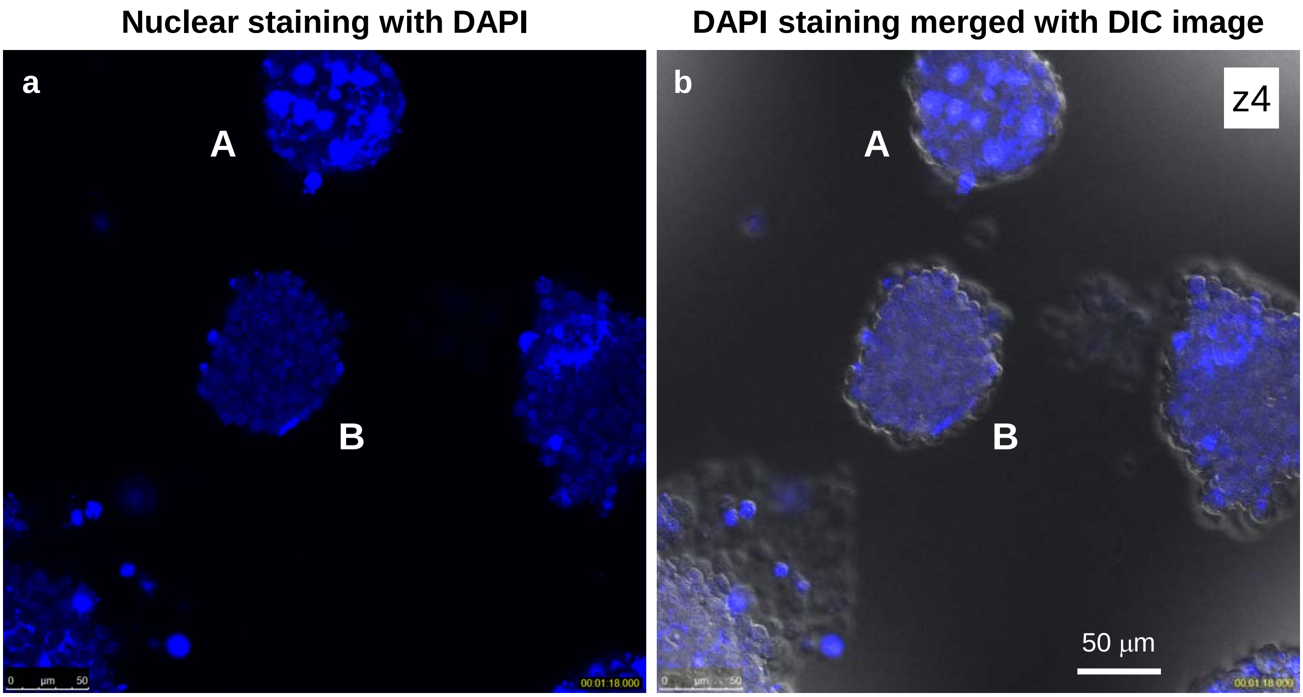

Online Resource 4-4. Similar to Online Resource 4-1, but image taken at 18 microns above.

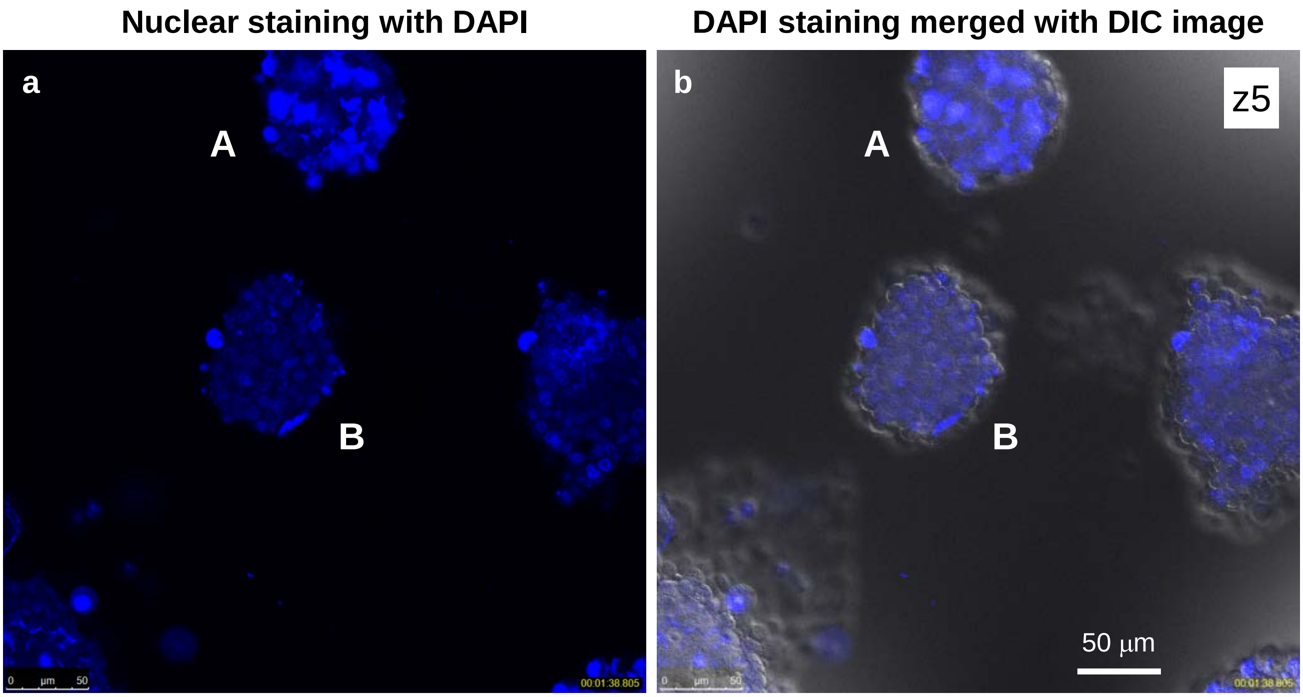

Online Resource 4-5. Similar to Online Resource 4-1, but image taken at 24 microns above.

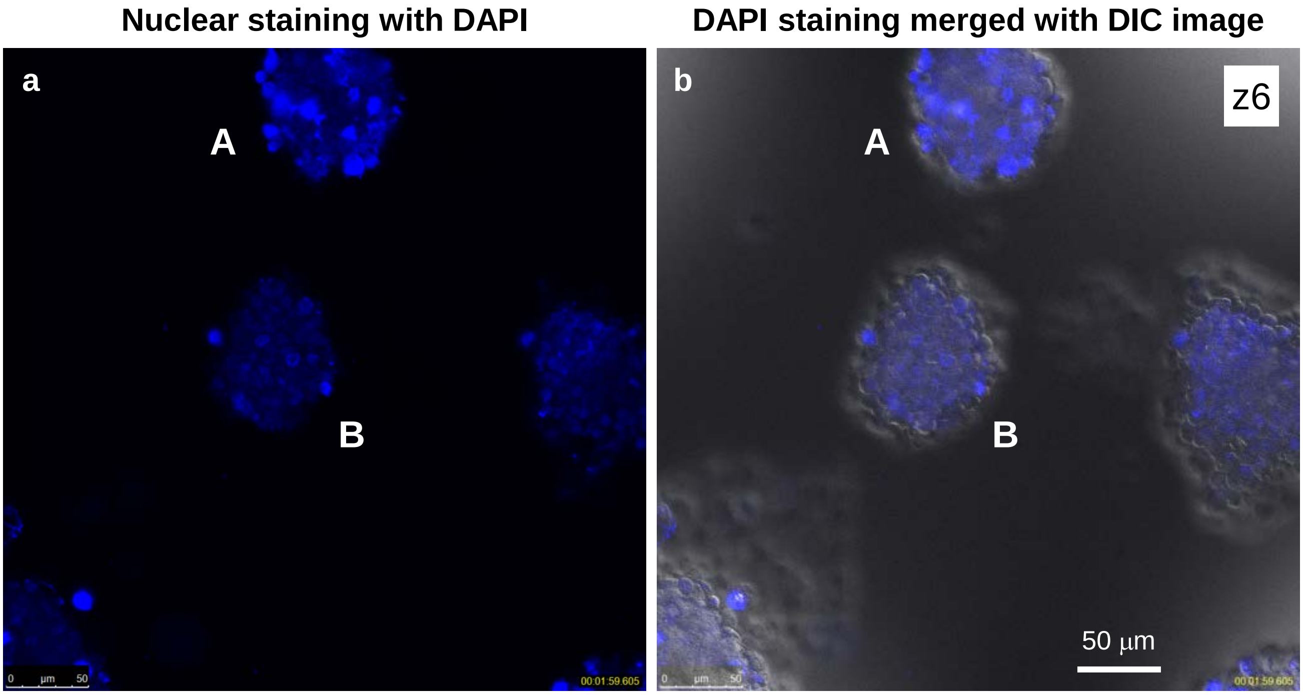

Online Resource 4-6. Similar to Online Resource 4-1, but image taken at 30 microns above.

Online Resource 5-1. Confocal microscopic images of 12 DIV MIN6 spheroids taken before starting the fluorescent tracer application, in three representative z sections. **a-f**, Pictures taken at the bottom part of the spheroid. **a**, Nuclear staining with DAPI in live cell condition. **b** and **c**, Fluorescence images taken in the green (b, 500-580 nm) and the red (c, 580-740 nm) wavelength range, reflecting entrance of 2-NBDLG and 2-TRLG, respectively. **d**, DIC image. **e**, Overlay of the green, red, and DIC images. **f**, Overlay of DAPI image and (e). **g-l**, and **m-r**, Similar to a-f, but images taken at 8 and 16 microns above, respectively. Bars are common to all panels.

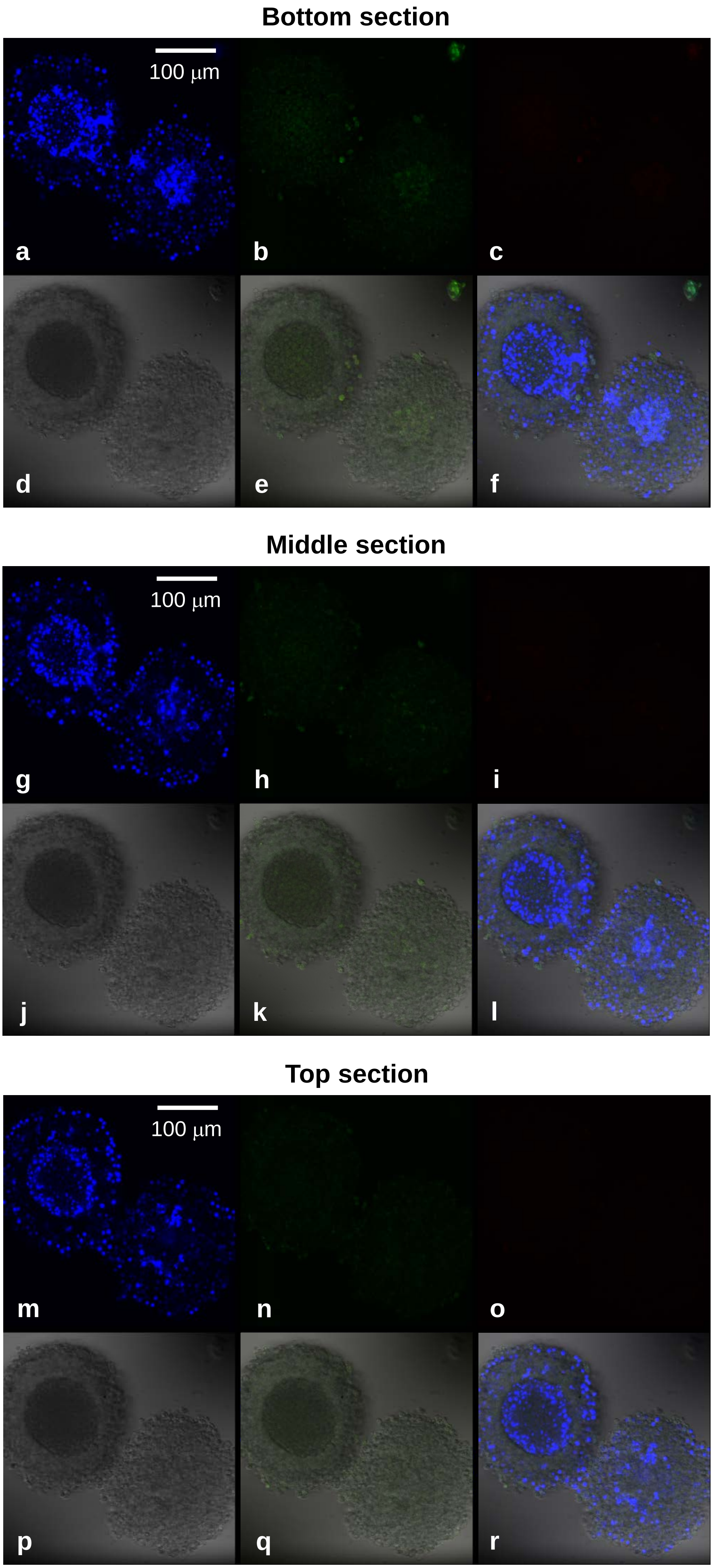

Online Resource 5-2.
Similar to Online Resource 5-1, but taken at 2 minutes after starting washout of 100 μM of 2-NBDLG (green) and 20 μM of 2-TRLG (red) mixture, in three representative z sections. **a-f**, Pictures taken at the bottom part of the spheroid. **a**, Nuclear staining with DAPI in live cell condition. **b** and **c**, Fluorescence images taken in the green (b, 500-580 nm) and the red (c, 580-740 nm) wavelength range, reflecting entrance of 2-NBDLG and 2-TRLG, respectively. **d**, DIC image. **e**, Overlay of the green, red, and DIC images. **f**, Overlay of DAPI image and (e). **g-l**, and **m-r**, Similar to a-f, but images taken at 8 and 16 microns above, respectively. Bars are common to all panels.

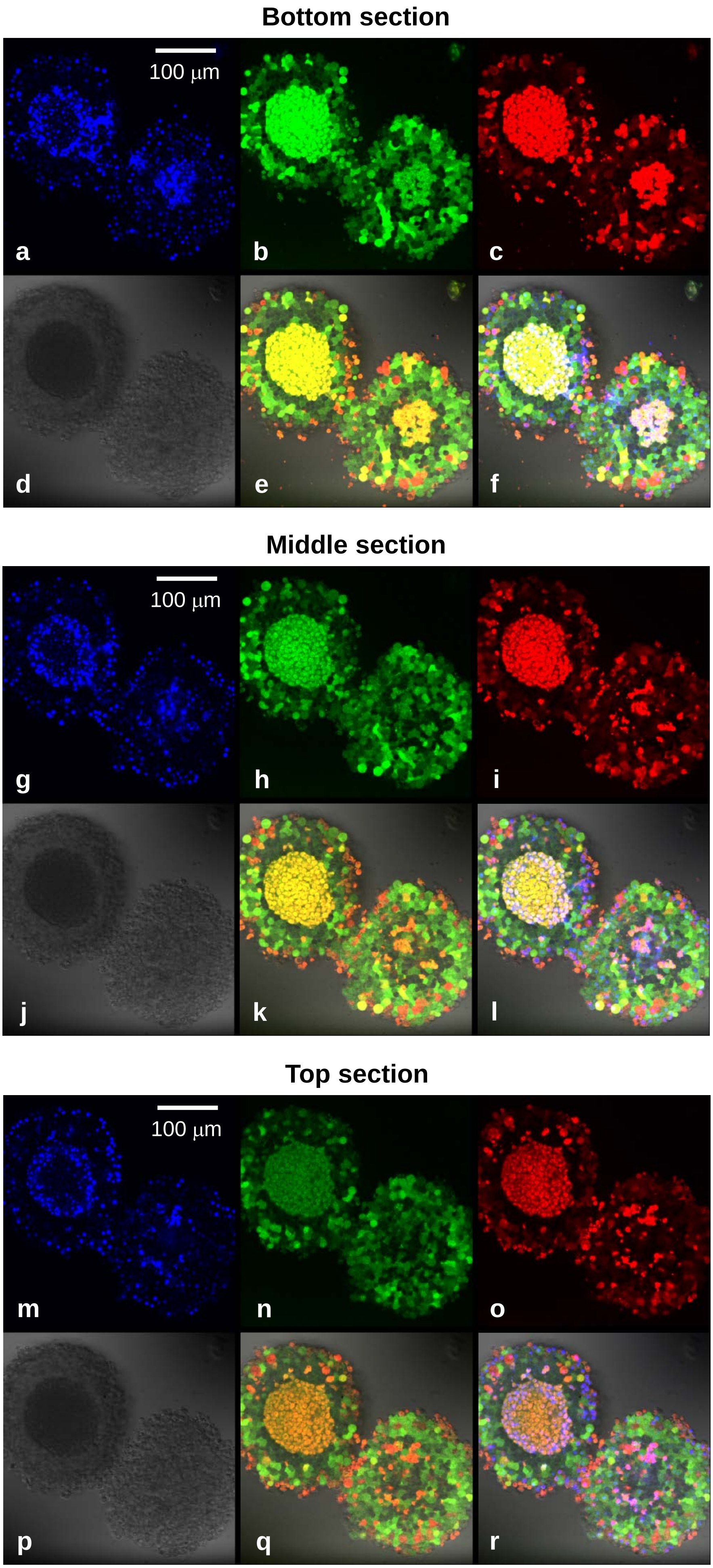

Online Resource 5-3. Similar to Online Resource 5-1, but taken at 4 minutes after starting washout of 100 μM of 2-NBDLG (green) and 20 μM of 2-TRLG (red) mixture, in three representative z sections. **a-f**, Pictures taken at the bottom part of the spheroid. **a**, Nuclear staining with DAPI in live cell condition. **b** and **c**, Fluorescence images taken in the green (b, 500-580 nm) and the red (c, 580-740 nm) wavelength range, reflecting entrance of 2-NBDLG and 2-TRLG, respectively. **d**, DIC image. **e**, Overlay of the green, red, and DIC images. **f**, Overlay of DAPI image and (e). **g-l**, and **m-r**, Similar to a-f, but images taken at 8 and 16 microns above, respectively. Bars are common to all panels.

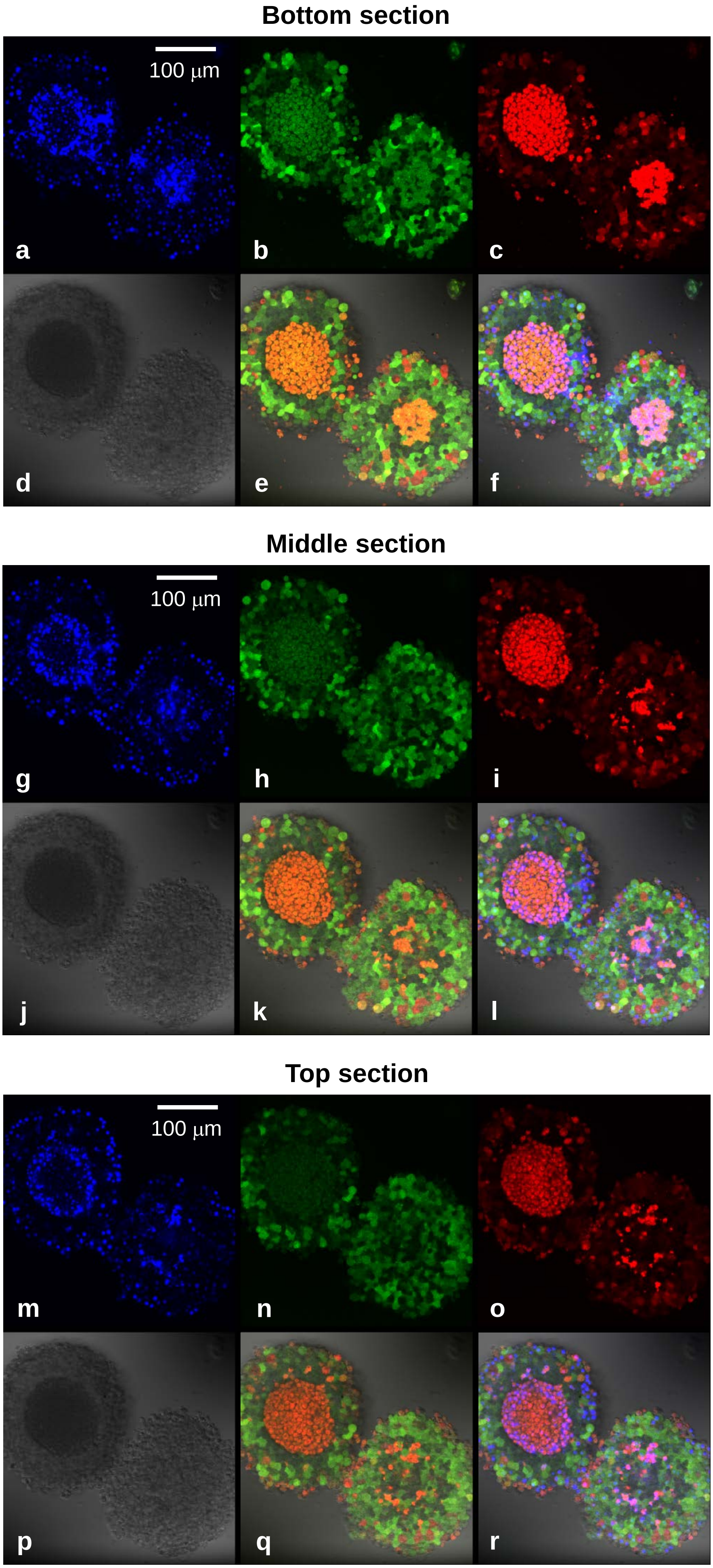

2-NBDG + 2-TRLG

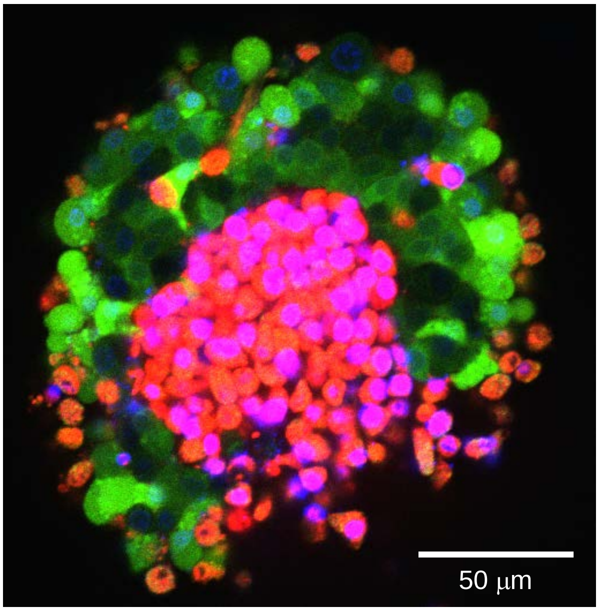

Online Resource 6. Confocal microscopic image of 13 DIV MIN6 spheroid taken at 4 minutes after starting washout of 100 μM of 2-NBDG (green) and 20 μM of 2-TRLG (red) mixture. Nuclei were stained by DAPI (blue). Note that dark and quiescent cells are somewhat less prominent in the surrounding area of the central core compared to those in Fig. 4l.

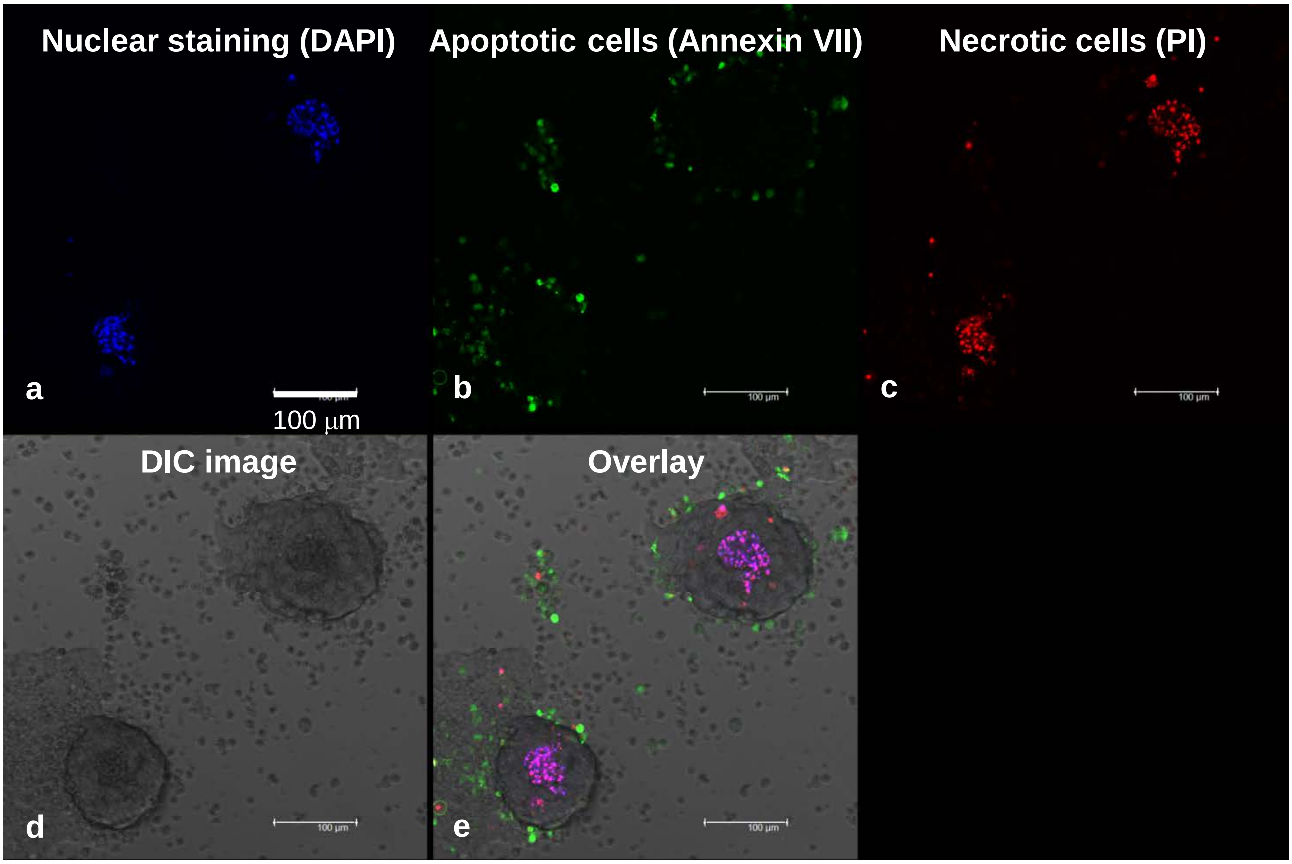

Online Resource 7. Representative MIN6 spheroids at 15 DIV including apoptotic and necrotic cells, each visualized by pSIVA-IANBD (green) and propidium iodide (PI, red), respectively, combined with DAPI staining during live imaging. **a**, Strongly DAPI-positive nuclei (blue) were seen mostly in the central core region of spheroids. **b**, Most pSIVA-IANBD-positive (green) apoptotic cells were seen in the outside of the spheroids. **c**, Most PI-positive (red) necrotic cells were localized in the central core of the spheroids. **d**, DIC image. **e**, Overlay. Scales are common to all panels.
